# Hardship in the Heartland: Associations Between Rurality, Income, and Material Hardship*

**DOI:** 10.1111/ruso.12435

**Published:** 2022-03-16

**Authors:** Aislinn Conrad, Megan Ronnenberg

**Affiliations:** ^1^ School of Social Work The University of Iowa

## Abstract

One in three U.S. households has experienced material hardship. The inadequate provision of basic needs, including food, healthcare, and transportation, is more typical in households with children or persons of color, yet little is known about material hardship in rural spaces. The aim of this study is to describe the prevalence of material hardships in Iowa and examine the relationship between rurality, income, and material hardship. Using data from the 2016 State Innovation Model Statewide Consumer Survey, we use logistic regression to examine the association between rurality, income, and four forms of material hardship. Rural respondents incurred lower odds than non‐rural respondents for all four hardship models. All four models indicated that lower income respondents incurred greater odds for having material hardship. Material hardship was reported across all groups, with rurality, income, race, and age as strong predictors of material hardship among our sample.

## Introduction

One in three U.S. households has experienced material hardship, with single mothers, children, and persons of color bearing a disproportionate burden of hardship (Conrad‐Hiebner and Paschall 2017; Rodems and Shaefer 2020). Material hardship is associated with a myriad of negative individual outcomes including depression, sleep problems, and increased stress levels (Huang et al. 2021), along with detrimental consequences for families. Parents with material hardship are more likely to rely on harsh parenting and experience child welfare involvement, leading to an increased likelihood of poor outcomes for children (Ashiabi and O’Neal 2007; Case et al. 2002; Conger and Donnellan 2007; Gershoff et al. 2007; Hernández et al. 2016; Huang et al. 2017; Kang 2013; Schenck‐Fontaine et al. 2020; Warren and Font 2015). Moreover, pregnant women who experience material hardship have increased risk of prenatal depression and anxiety, which may have serious effects on the health of unborn children (Katz et al. 2018). Despite our knowledge of these consequences, we still have limited understanding of the prevalence of material hardship in some populations, including those who live in rural spaces. The purpose of this study is to examine the incidence and covariates of material hardship among rural and non‐rural households in Iowa. Specifically, we focus on the relationship between income and rurality with material hardship. Our study contributes to both empirical and theoretical literature on material hardship, demonstrating the likelihood of experiencing hardship in rural and non‐rural communities along with the relationship between income and material hardship or *economic hardship*.

Historically, researchers have not investigated the incidence of economic hardship among rural households (Myers and Gill 2004), despite higher rates of poverty among rural households as compared to non‐rural (Schaefer et al. 2016). This is problematic given what we know about daily life in rural spaces. For example, rural areas are less densely populated than urban areas, meaning that residents travel longer distances to daily activities (Ratcliffe et al. 2016). Furthermore, *rural decline*, or the decreasing populations and deteriorating employment opportunities in rural communities, means that some rural residents face increasing barriers to procure jobs and subsequently basic needs (Li et al. 2019). Without understanding the extent of material hardship in rural households, rural families may remain at risk for the social and economic consequences of material hardship.

### Economic Hardship: Income and Material Hardship

Both income and material hardship are distinct dimensions of *economic hardship* (Conrad et al. 2020; Conger et al. 1992; Gershoff et al. 2007; Schenck‐Fontaine and Panico 2019), yet most researchers only measure income (Heflin 2017). Whereas measures of *income* depict the financial resources available to households, measures of *material hardship* demonstrate whether households can meet their basic needs for goods and services like food, transportation, housing, and medical care (Beverly 2001; Fusco et al. 2011). Further, some researchers argue that measures of material hardship are more objective measures of economic wellbeing than income because material hardship indicators assess households' use of goods and services rather than cash flow (Beverly 2001; Conrad‐Hiebner and Scanlon 2015). Until we include material hardship as indicators of economic hardship, researchers cannot fully describe economic hardship among households.

The conceptual differences between income and material hardship also have empirical support. Low income and material hardship share a weak association (Mayer and Jencks 1989), with only 6 percent of respondents experiencing simultaneous housing and bill pay hardship and poverty according to one UK study (Schenck‐Fontaine and Panico 2019). However, Conrad and colleagues (2019) found that 36 percent of US households had food hardship and 6 percent of households had healthcare access hardship among a national sample of low‐income urban mothers. Though low‐income households are more likely to face material hardship, higher‐income households are also at risk (Bradshaw and Finch 2003; Iceland and Bauman 2007; Lee and Lee 2016; Neckerman et al. 2016). In addition to income, factors like financial literacy, regional cost of living, childcare costs, student loan debt, medical bills and unanticipated expenses may elucidate why some households can purchase food and rent while others cannot (Beverly 2001; Lusardi 2008; Mayer and Jencks 1989, 1993; Ouellette et al. 2004).

There are two primary conceptual frameworks that examine how economic hardship impacts households, including the family investment model and the family stress model. In the family investment model (Conger and Donnellan 2007), household income allows parents to transfer goods and services like childcare and educational opportunities to their children, which increases child academic achievement and promotes warmer parenting practices (Berger and Font 2015; Bradley and Corwyn 2002; Conger et al. 1992; Duncan and Magnuson 2003; Schofield et al. 2011; Sohr‐Preston et al. 2013; Yeung et al. 2002). Whereas income is the main dimension of economic hardship in the family investment model, both income and material hardship are part of the family stress model (Conger et al. 1992). Both low income and material hardship exacerbate parental stress and depression (Conrad‐Hiebner and Scanlon 2015; Conger et al. 1992; Eamon 2001; Newland et al. 2013; Yang 2015; Yeung et al. 2002).

There is some evidence that material hardship and income differentially impact household outcomes. Regardless of household income levels, material hardship is related to poor behavior, physical health and social‐emotional competence in children (Gershoff et al. 2007; Lee and Lee 2016; Paat 2011; Zilanawala and Pilkauskas 2012). Further, material hardship appears to influence parenting behaviors and parenting stress whereas income effects parents’ investments in their children's development. The differential impacts of income and material hardship perhaps explain why income is associated with child cognitive development and material hardship is related to child mental health (Gershoff et al. 2007).

In all, income and material hardship represent two distinct, though related, components of economic hardship with negative consequences for households. Given the contributions of both income and material hardship to economic hardship, we include both measures as variables in our study.

### Rurality and Material Hardship

The experience of material hardship may vary by geographic location and regional or local culture (Karpman et al. 2018). Residents in the Southern US region have higher odds of health, food, and bill‐pay hardship compared to those in the Northeast region, and urban residents have higher odds of bill‐pay and food hardships compared to non‐urban (Iceland, Kovach, and Creamer 2021). However, only a few studies have examined the distribution of material hardship between rural and non‐rural residents (e.g., Despard et al. 2021; Heflin 2017; Karpman et al. 2018), and the relationship between income and material hardship among rural populations remains unknown (Conrad et al. 2019; Gershoff et al. 2007; Mayer and Jencks 1989; Schenck‐Fontaine and Panico 2019). Despard and colleagues (2021), for example, found that rural residents were less likely than urban to experience food hardship, but their sample only included low‐income households. The purpose of this study is to examine the effect of rurality and income on material hardship among all Iowa households, a state with a large rural population.

Rurality is typically conceptualized based on the size and population density of a geographic area, primary types of land use (e.g., agriculture), and shared culture tied to living on an expansive landscape (Cloke 2006; Nelson et al. 2021). Some scholars suggest that rurality is a fluid, socially constructed concept dependent on individual perceptions, experiences and cultural differences (Nelson et al., 2021) rather than an objective measure of place (Tickamyer 2020). That is to say, there is great heterogeneity among rural communities which could influence the experience of material hardship. Rural areas with strong agriculture economies, like most of Iowa, are culturally distinct from chronically poor rural areas where residents are accustomed to hard economic times (Ulrich‐Schad and Duncan 2018). Whereas relying on social assistance (e.g., food stamps) to cover household necessities may be culturally acceptable or even expected in chronically poor rural areas, those in areas with historically strong agriculture economies may be less likely to apply for assistance programs due to cultural stigma (Brooks and Voltaire 2020; Cramer 2016; Edelman 2021; Thiede et al. 2016; Ulrich‐Schad and Duncan 2018)). Cultural nuances may influence the experience of material hardship, and it is important for researchers to examine material hardship and its predictors across a variety of rural settings. Our unique data set offers an opportunity to examine material hardship in a population that is relatively affluent compared to some other states with large rural populations.

### The Present Study

Due to a lack of literature on material hardship in rural households, the underutilization of rurality as a predictor of material hardship, and varying estimates of material hardship, we examined the relationship between rurality, transportation hardship, food hardship, healthcare access hardship, and any material hardship among a representative sample of adults residing in Iowa. Our study will draw attention to the prevalence of economic hardship of rural households, contribute to our understanding of the association between income and material hardship, and demonstrate associations between forms of hardship.

In all, our paper contributes to the literature by examining the association between income and four forms of material hardship in both rural and non‐rural households, and the relationship between forms of material hardship. Policymakers, advocates, and community leaders can use our findings when advocating for more resources within their rural communities. We hypothesized that 1) rural households would have increased odds for experiencing material hardships than non‐rural households, and 2) lower‐income households would incur greater odds for having material hardships than higher‐income households.

## Method

### Study Design

We examined associations between household income, rurality, transportation hardship, food hardship, healthcare access hardship, and any hardship using data from the 2016 Iowa State Innovation Model (SIM) Statewide Consumer Survey (SCS) (*N* = 2,132) after being approved by our institutional review board. Through dual‐frame random digit dial sampling procedures with landline and cellular phones, SIM researchers conducted Computer Assisted Telephone Interviewing (CATI) between September 2016 and April 2017 with adult Iowans 18 years and older about their physical health, mental health, and healthcare use as part of a broader evaluation of the State of Iowa’s implementation of healthcare innovations. The overall response rate was 27.1 percent with a cooperation rate of 73.5 percent. The SCS data is representative of all adult Iowans after applying weights that account for SCS’s sampling strategies and differential nonresponse (Bentler et al. 2017). Prior to analysis, we examined the percentage of missingness, which ranged from 0 percent to 12.7 percent for the study variables. We did not impute data for variables due to the small amount of missingness (Bennett 2001).

### Measures

#### *Material* *hardship*

The literature lacks consensus on how to measure material hardship or what hardships to include (Heflin et al. 2009; Ouellette et al. 2004), with some researchers assessing both individual and overall hardship (Osborne et al. 2012; Pilkauskas et al. 2012). We followed Pilkauskas and colleagues' (2012) approach for estimating forms of material hardship separately and using a summary measure. In Appendix A, we detail our approach for constructing three indicators of material hardship, including transportation hardship, healthcare access hardship, and food hardship, along with a summary measure for any material hardship. We dichotomized our indicators of material hardship, with scores of “1” indicating that respondents reported a hardship in the last year, and scores of “0” indicating the absence of that type of hardship.

We estimated food hardship using the Food Security Module Short‐Form (Blumberg et al. 1999), which is used widely by researchers (e.g., Conrad‐Hiebner and Paschall 2017; Conrad et al. 2019; Yang 2015). The scale consists of five items indicating whether and to what extent respondents experienced types of food hardship. Respondents who reported experiencing at least one of the five types of food hardship at least “sometimes” were coded as “1” for having food hardship. We used four measures to determine healthcare access hardship: two frequently used indicators (absence of health insurance coverage and inability to see the doctor when needed) along with two measures related to transportation to healthcare visits (see Appendix) (Conrad‐Hiebner and Scanlon 2015). The questions about transportation to healthcare visits asked whether there was any time in the past 12 months *when you needed transportation to or from a health care visit but could not get it for any reason* (yes or no) and how often in the past 12 months did you worry *about your ability to pay for the cost of transportation to or from a health care visit* (never, sometimes, usually, or always).

Transportation hardship is rarely included in studies of material hardship despite demonstrated associations between car ownership and well‐being related to employment, housing, or neighborhood quality (Bastiaanssen et al. 2020; Blumenberg and Pierce 2017; Jeon et al. 2018). Although car ownership may not perfectly capture transportation access, *car deficit* (i.e., less than one car per adult per household) is a proxy of scarce household resources (Blumenberg et al. 2020) that is more often due to financial constraints than personal choice (Brown 2017; Klein and Smart 2017). Car deficit households, particularly those that are also low‐income, must negotiate the use of household vehicles when other forms of transit are too expensive or not available. Given this bearing of personal vehicles on transportation, households demonstrated *transportation hardship* if they had less than one vehicle available per adult driver (“1”) (Blumenberg et al. 2020).

#### *Independent* *variables*

The data set included a subjective indicator of rurality based on the survey question, “Which of the following best describes the area where you live?”, where response options were (1) on a farm, (2) in a rural setting but not on a farm, (3) in a rural subdivision outside of city limits, (4) in a small town of less than 5k people, (5) in a large town of 5k to less than 25k people, (6) in a city of 25k to less than 50k people, (7) in a city of 50k to less than 150k people, or (8) in a city of 150k or more. We computed a dichotomous measure, with respondents who reported living on a farm, in a rural area, or a rural subdivision outside of city limits coded as rural (“1”) and everyone else as non‐rural (“0”). This is a unique measure that differs from standard ways to delineate rurality, which typically rely on official estimates of population size and density (e.g., Metropolitan Statistical Area (MSA) codes and the US Census Bureau’s urbanized areas and urban clusters classifications).

Income was measured using five categories in a question designed by the survey developers in 2016: (1) < $25,000, (2) $25,000–< $50,000, (3) $50,000–< $75,000, (4) $75,000–< $100,000, and (5) $100,000 or more. We created four dummy variables for income, with the final category, $100,000 or more, as the reference category for our logistic regression models.

### Statistical Approach

First, we describe our sample using both descriptive statistics and chi‐square tests to examine the differences between respondents with any material hardship and respondents without material hardship by income, rurality, and covariables. Variables that showed a statistically significant bivariate relationship with hardship were included in the next phase of analysis, binary logistic regression. We used four logistic regression models to investigate the odds of having any material hardship, healthcare access hardship, transportation hardship, and food hardship in STATA (2019) (see Table 2). Covariates included gender, race, age, education, employment status, and children in the household. We chose to include a dichotomous indicator for whether children resided in the household in place of a measure for household size because the number of adults in the household was included in the transportation hardship measure.

## Results

### Sample Characteristics

Weighted and unweighted frequencies are reported in Table 1. Notably, only 20 percent of the sample identified as rural, compared to the US Census Bureau estimate of 36 percent (ISU 2010). Two‐fifths of respondents experienced one or more material hardships (40 percent), with more respondents reporting food hardship (26 percent) than healthcare access hardship (20 percent) and transportation hardship (15 percent).

**Table 1:** Descriptive Statistics for Study Variables (Weighted *N* = 2,371,739)

Variable	Unweighted *N* (%)	Weighted *N* (%)	Iowa‐ US Census Bureau Est.	USA‐ US Census Bureau Est.
Place of Residence				
Rural	592 (28.7)	456,486 (20.0)	35.98%	19.30%
Non‐Rural	1,474 (71.3)	1,826,966 (80.0)	64.02%	80.7%
Income				
Under 25K	418 (19.6)	296,982 (12.5)	10.19%	13.79%
25–49K	520 (24.4)	534,898 (22.6)	20.37%	20.64%
50–74K	417 (19.6)	427,337 (18.0)	21.51%	18.40%
75–99K	327 (15.3)	354,539 (14.9)	17.62%	14.49%
100K ≤	450 (21.1)	757,983 (32.0)	30.32%	32.72%
Race				
Non‐White	184 (8.6)	298,629 (12.6)	9.10%	26.66%
White	1,948 (91.4)	2,073,110 (87.4)	90.90%	73.35%
Gender				
Female	1,055 (50.5)	1,203,703 (50.8)	50.33%	50.79%
Male	1,055 (49.5)	1,168,036 (49.2)	49.67%	49.21%
Age				
18–24	152 (7.1)	278,125 (11.7)	13.46%	12.77%
25–34	294 (13.8)	429,189 (18.1)	16.44%	17.71%
35–44	268 (12.6)	372,301 (15.7)	15.32%	16.55%
45–54	360 (16.9)	384,084 (16.2)	17.07%	17.74%
55–64	478 (22.4)	421,298 (17.8)	17.06%	16.35%
65 ≤	580 (27.2)	486,742 (20.5)	20.62%	18.85%
Education				
High School ≥	668 (31.3)	931,379 (39.3)	40.16%	40.55%
Some College	735 (34.5)	760,912 (32.1)	32.68%	29.14%
4‐Year Degree ≤	729 (34.2)	679,448 (28.6)	27.17%	30.32%
Employment Status				
Unemployed	811 (38.0)	788,847 (33.3)	21.39%	28.84%
Employed	1,321 (62.0)	1,582,892 (67.7)	78.61%	71.16%
Children in Household				
Yes	622 (29.2)	886,076 (37.5)	43.72%	43.25%
No	1,507 (70.7)	1,479,288 (62.5)	56.28%	56.75%
Food Hardship				
Yes	555 (26.0)	624,778 (26.4)	–	–
No	1,575 (73.9)	1,744,068 (73.6)	–	–
Healthcare Access Hardship				
Yes	407 (19.1)	473,763 (20.0)	–	–
No	1,725 (80.9)	1,897,976 (80.0)	–	–
Transportation Hardship				
Yes	282 (13.3)	357,364 (15.1)	–	–
No	1,844 (86.7)	2,006,017 (84.9)	–	–
Any Hardship				
Yes	813 (38.1)	942,789 (39.8)	–	–
No	1,319 (61.9)	1,428,950 (60.2)	–	–

Respondents in the lowest income group were most likely to report any material hardship, and each subsequent income group was less likely to report material hardship than the preceding group (Table 2). We observed this statistically significant negative relationship between income and material hardship in chi‐square analysis between income and each form of material hardship. For example, 34 percent of respondents in the lowest income group reported transportation hardship, compared to only 5 percent in the highest income group.

**Table 2:** Chi‐Square Results for Study Variables and Any Hardship

Variable	No Hardship	Any Hardship
	Weighted *N* (%)	Weighted *N* (%)
Place of Residence		
Rural	317,503 (70)	138,983 (30)
Non‐Rural	1,085,755 (59)	741,211 (41)
Income		
Under 25K	70,028 (24)	226,954 (76)
25–49K	241,087 (45)	293,811 (55)
50–74K	237,158 (55)	190,179 (45)
75–99K	254,365 (72)	100,174 (28)
100K ≤	626,312 (83)	131,671 (17)
Race		
Non‐white	86,066 (29)	212,563 (71)
White	1,342,884 (65)	730,226 (35)
Gender		
Female	701,609 (58)	502,094 (42)
Male	727,341 (62)	440,695 (38)
Age		
18–24	103,005 (37)	175,120 (63)
25–34	207,910 (48)	221,279 (52)
35–44	234,181 (63)	138,120 (37)
45–54	248,217 (65)	135,867 (35)
55–64	289,621 (69)	131,677 (32)
65 ≤	346,015 (71)	140,727 (29)
Education		
High School ≥	432,567 (46)	498,812 (54)
Some College	478,821 (63)	282,091 (37)
4‐Year Degree ≤	517,562 (76)	161,886 (24)
Employment Status		
Unemployed	434,658 (55)	354,189 (45)
Employed	994,292 (63)	588,600 (37)
Children in Household		
Yes	474,939 (54)	411,138 (46)
No	950,748 (64)	528,540 (36)

Note:

All differences are statistically significant at *p* < .05.

Respondents reporting any material hardship were also more likely to live in non‐rural areas, to report being young (ages 18–24), or to identify as persons of color than respondents without hardship. Conversely, respondents without hardship were most likely to be older (age 65+), earn annual incomes of $75,000 or more, live in rural areas, and have college degrees.

### Logistic Regression Models

In Table 3, we present results from our four logistic regression models testing the relationship between rurality, income, food hardship, healthcare access hardship, transportation hardship, and any hardship. None of the models supported our first hypothesis on the relationship between rurality and hardship: Rural respondents had lower odds of all forms of hardship than non‐rural respondents. However, all models provided support for our second hypothesis on the association between income and hardship: Lower income respondents incurred greater odds of all forms of hardship than higher income respondents.

**Table 3:** Odds Ratios for Hardship Models

Variable	Any		Healthcare		Transportation		Food	
	ß (SE)	OR	ß (SE)	OR	ß (SE)	OR	ß (SE)	OR
Rural	−0.23 (.004)	0.79	−0.03 (.004)	0.97	−0.34 (.005)	0.71	−0.22 (.004)	0.80
Income								
Under 25K	2.27 (.006)	9.75	1.89 (.007)	6.65	1.36 (.007)	3.90	2.58 (.006)	13.20
25K–49K	1.39 (.005)	4.01	1.41 (.006)	4.09	1.29 (.006)	3.62	1.61 (.005)	5.02
50K–74K	1.06 (.005)	2.89	0.87 (.006)	2.38	0.66 (.007)	1.93	1.40 (.005)	4.04
75K–99K	0.24 (.005)	1.27	0.43 (.007)	1.53	‐0.10 (.009)	0.91	0.45 (.006)	1.57
100K ≤ (ref)	–	–						
Non‐white	0.92 (.005)	2.51	0.80 (.005)	2.23	0.93 (.005)	2.54	0.25 (.005)	1.28
Female	0.04 (.003)	1.04	0.004 (.003)	1.00*	0.25 (.004)	1.28	0.04 (.003)	1.04
Age								
18–24	1.49 (.006)	4.44	1.31 (.007)	3.71	0.87 (.007)	2.39	1.39 (.006)	4.03
25–34	1.11 (.006)	3.05	1.32 (.007)	3.76	0.79 (.007)	2.22	1.63 (.006)	5.10
35–44	0.60 (.007)	1.83	1.26 (.007)	3.52	0.09 (.008)	1.09	1.34 (.007)	3.81
45–54	0.68 (.006)	1.98	0.86 (.007)	2.36	0.44 (.007)	1.55	1.21 (.006)	3.36
55–64	0.36 (.006)	1.43	0.96 (.006)	2.60	‐0.02 (.007)	0.98	0.78 (.006)	2.18
65 ≤ (ref)	–	–	–	–				
Education								
≤ High School	0.62 (.004)	1.86	0.59 (.005)	1.80	0.45 (.006)	1.56	0.60 (.005)	1.82
Some College	0.24 (.004)	1.28	0.22 (.005)	1.25	0.01 (.006)	1.01	0.44 (.005)	1.55
4‐Year Degree ≤ (ref)	–	–						
Unemployed	0.30 (.004)	1.35	0.35 (.004)	1.41	0.71 (.004)	2.03	0.39 (.004)	1.48
Children in the Home	0.41 (.004)	1.50	0.26 (.004)	1.29	0.02 (.005)	1.02	0.35 (.004)	1.42
*Pseudo R^2^*	.1876		.1499		.1422		.1839	

* Indicates a non‐statistically significant association. All other associations are statistically significant at *p* < .05.

#### *Any* *hardship*

**Figure 1:**
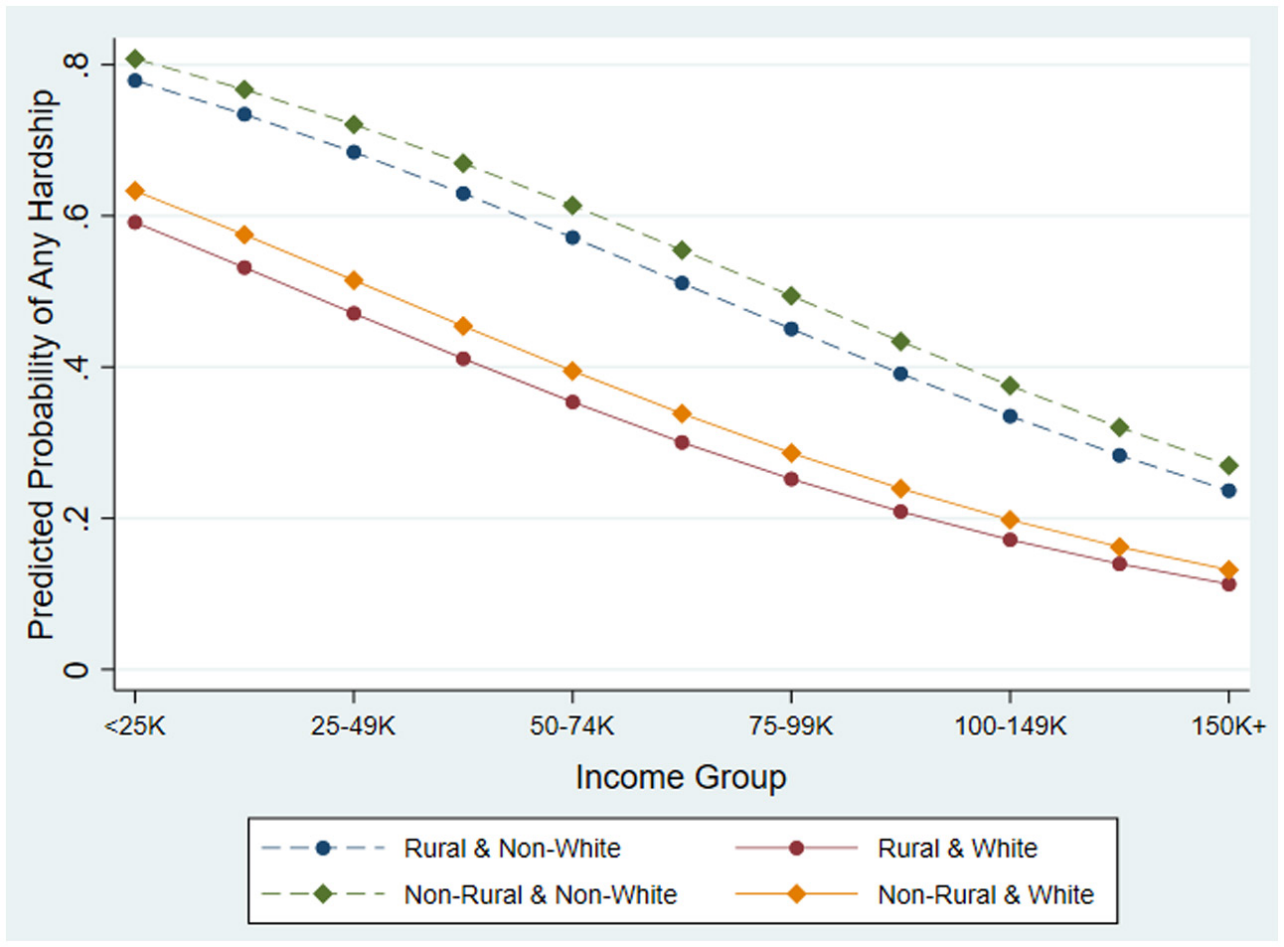
Predicted Probabilities of Any Hardship by Income and Rurality.

Non‐rural respondents and those reporting the lowest income, a non‐White racial status, or the youngest age sustained increased odds for having any hardship. As seen in Figure 1, Rural respondents were 21 percent less likely than non‐rural to experience any hardship and non‐White respondents had 2.5 times the odds for experiencing any hardship compared to White. Most striking is the difference between the lowest and highest income groups; respondents earning less than $25,000 had nearly 10 times the odds for experiencing any hardship than the highest‐income group, and the magnitude of the odds decreased considerably as income increased.

There were also differences by age, education, and households with children. Young respondents (ages 18–24) had 4.5 times higher odds than the oldest age group for reporting any hardship, with the magnitude of the odds decreasing as age increased. Respondents with children in the home or an education of high school or less had higher odds of reporting any hardship than college‐educated respondents or respondents with no children in the home.

#### *Healthcare* *access hardship*

Living in a rural area decreased respondent odds of healthcare access hardship by about three percentage points. Income, age, and race shared strong associations with healthcare access hardship. For example, respondents in the lowest income group had about 6.65 times higher odds of healthcare access hardship compared to those in the highest income group, and like any hardship, the magnitude of the odds decreased for each subsequent income group. The odds ratios for age groups followed a pattern similar to income, where the youngest age group had considerably higher odds for healthcare access hardship compared to the oldest age group, and the magnitude of the odds decreasing with each age group. Non‐white respondents incurred 2.23 times higher odds for healthcare access hardship compared to White.

#### *Transportation* *hardship*

Respondents living in rural areas were 29 percent less likely to experience transportation hardship compared to those in non‐rural areas. Income had a nonlinear relationship with transportation hardship. Those earning between $25,000 and $49,999 had 3.62 times higher odds of transportation hardship compared to those in the highest income group, whereas respondents earning $75,000–$99,000 were 9 percent less likely to have transportation hardship as compared to those earning $100,000 or more (see Table 3). The odds ratios for age groups followed a pattern similar to income. The youngest age group had 2.39 times higher odds of transportation hardship compared to the oldest age group, while those in the 55–64 age group were less likely to incur transportation hardship compared to those 65 or older. Respondents reporting as female or non‐White incurred higher odds for experiencing transportation hardship as compared to males or White respondents.

#### *Food* *hardship*

As compared to non‐rural respondents, rural respondents were 20 percent less likely to demonstrate food hardship. Race and age also shared strong associations with food hardship, whereas the impact of gender on food hardship was negligible (see Table 3). Respondents of every income level and age group had higher odds for food hardship than respondents in the reference groups. The lowest‐income earners were nine times more likely to experience food hardship than the highest‐income earners. Finally, respondents reporting ages 25 to 34 or incomes of $50,000 to $75,000 had more than five times higher odds of food hardship compared to the oldest and highest income groups, respectively.

#### *Summary* *of findings*

Results from the logistic regression indicated that rural residents had lower odds for experiencing all forms of hardship. Respondents in all income groups had higher odds of hardships compared to the highest income groups, and 17 percent of respondents in the highest income group reported any material hardship.

## Discussion

According to the literature, both material hardship and rural poverty negatively impact personal wellbeing (Huang et al. 2017; Schaefer et al. 2016; Schenck‐Fontaine et al. 2020), yet little is known about the association between rurality and material hardship or between material hardship and income in rural U.S. households. In the absence of this knowledge, we cannot determine the unique influence of rurality on the experience of material hardship among Midwestern‐dwelling households. In response, we described the prevalence of rurality, income, and four hardship types, and conducted chi‐square difference testing and logistic regression to examine the relationship between rurality, income, food hardship, transportation hardship, healthcare access hardship, and any hardship. We found support for the following themes: (1) Though material hardship occurs in households with all income levels, lower‐income households have higher odds of hardship than higher income; (2) rurality decreased the odds for all hardships; and (3) age and race are also strong predictors of material hardship in our sample, with young and non‐White individuals at highest risk. Taken together, our findings contribute to the literature on material hardship.

Material hardship was pervasive in our study, with 40 percent of respondents reporting at least one hardship related to food, healthcare access, or transportation. Our findings suggest that material hardship may be more prevalent in some areas of the U.S. than formerly reported. Previous estimates with national samples indicated 14 percent to 36 percent of respondents with one or more hardships (Heflin 2016; Rodems and Shaefer 2020; Schenck‐Fontaine and Panico 2019). Surveillance issues (e.g., low‐income samples versus nationally‐representative samples) and measurement differences most likely contribute to the contrasting estimates of material hardship and have been thoroughly discussed elsewhere (Conrad‐Hiebner and Byram 2020; Heflin 2006; Ouellette et al. 2004). In the following paragraphs, we discuss how our use of a Midwestern sample and an expanded measure of material hardship may have contributed to our estimates on material hardship.

First, our unique sample may partially explain why material hardship is more prevalent in our study compared to others. We engaged a representative sample of households from Iowa, a rural state, which is not common in literature on material hardship in the U.S. Most U.S. studies on material hardship rely on samples of urban or low‐income households (e.g., Heflin 2006), which may not represent households from rural states. Second, like other researchers studying material hardship, we measured food and health insurance hardship (Ouellette et al. 2004).

In contrast to the extant literature, we expanded our inquiry to include transportation hardship and healthcare access hardship, which are understudied in literature on material hardship. Our decision to include additional hardships may have increased the number of households with hardship in our study. Further, decisions about how to count material hardship may have impacted our estimates. We operationalized food hardship as the presence of one or more indicators from Blumberg and colleagues' (1999) Household Food Security Survey, for example, while another research team defined food hardship as the presence of two or more indicators from the same survey (Rodems and Shaefer 2020). Though our calculation of material hardship is more liberal than those in Rodems and Shaefer's (2020) study, our operationalization closely aligns with other literature on material hardship (Conrad et al. 2019; Pilkauskas et al. 2012; Schenck‐Fontaine et al. 2017). These discrepancies in measurement indicate a need for standardized measures of material hardship, which are in initial stages of development (e.g., Conrad 2020). Standardized measures would help researchers compare rates of material hardship across various samples and settings.

Comparable to other studies, material hardship was more frequent among respondents reporting young ages, children in the home, status as persons of color, or low socioeconomic status (Mayer and Jencks 1989; Rodems and Shaefer 2020). Most low‐income respondents experienced material hardship (76 percent) in our study, with odds for all hardship types increasing as age, income, educational attainment and employment decreased. Interestingly, though our lowest‐income respondents experienced the most material hardship, higher earners also reported material hardship. Nearly 20 percent of high‐income respondents ($100,000+) had material hardship, with those in the second highest‐income group ($75,000–99,000) incurring 1.27 to 1.51 higher odds for any hardship, food hardship, and healthcare access hardship. Our findings align with other researchers who documented the presence of material hardship among non‐poor households (Iceland and Bauman 2007; Mayer and Jencks 1989; Short 2005).

There are multiple factors that may clarify why higher‐income households have material hardship. Income describes one dimension of economic hardship, yet on its own cannot guarantee that households are adequately fed, housed, and clothed. Along with a lack of financial literacy, there are differences in cost‐of‐living, debt and savings, fluctuations in health insurance coverage, and unanticipated expenses that contribute to household financial uncertainty (Beverly 2001; Lusardi 2008; Mayer and Jencks 1989). Nearly 85 percent of U.S. households, for example, carry debt (Haughwout and van der Klaauw 2015), with 25 percent of middle‐income families unable to secure US$2000 for unexpected purchases (Lusardi et al. 2011).

Despite the prevalence of material hardship, income poverty rather than material hardship drives eligibility guidelines for U.S. social safety net programs providing cash and food assistance. Social safety net programs are designed to help households afford food, housing, and medical care, yet only target low‐income households. In a recent study of material hardship in the U.S. between 1991 and 2011, Iceland and colleagues (2021) found that material hardship declined over the study period for low‐income households, likely due to an increase of noncash benefits like food assistance and subsidized insurance. A limitation of our data is that it does not include an indicator for receipt of assistance or noncash benefits. Once household income matches or doubles the financial threshold determined by federal poverty guidelines, U.S. households likely do not qualify for assistance through social safety programs (DHHS 2020).

Although some might argue that higher income households do not require assistance through social safety net programs, our results on the relationship between middle‐income households (US$50,000–$74,000) and material hardship suggest otherwise. Though median household income of US$63,179 exceeded federal poverty guidelines in 2018 (US$25,100) (USCB 2019), this income level falls below the US$67,146 estimated to meet day‐to‐day necessities for a family of four (Nadeau and Glasmeier 2019). The difference between median income and the living wage calculator (Glasmeier and Massachusetts Institute of Technology 2020) indicates a gap between the intended and actual recipients of social safety net programs. In other words, a number of middle‐income households demonstrating material hardship may not receive the assistance they need.

In addition to describing the incidence of material hardship, our study is one of the first to estimate material hardship for rural households in Iowa. Our results indicated a buffering impact of rurality on material hardship: Rural households had lower odds than non‐rural households in all four models of hardship. These findings contextualize rural economic hardship, that is, the combined impact of economic resources (income) and lived experience of financial uncertainty (material hardship) on households (Schenck‐Fontaine and Panico 2019). Given these findings, it is possible that rural households have less overall economic hardship than non‐rural households. However, researchers need to further test this relationship across a variety of rural settings using measures that capture multiple dimensions of rurality. Findings from Iowa may differ from rural areas that are less dependent on agriculture, are less or more affluent, or have cultural differences.

Though there is limited literature on rural material hardship, recent data on perceptions regarding safety net programs and trend data on rural poverty supports our tentative hypothesis: Nolan and colleagues (2017) found that rural poverty dramatically declined from 1967 to 2014 as compared to urban poverty because social safety net programs have strengthened in rural areas. In another study (Haynes‐Maslow et al. 2020), rural respondents with food insecurity reported the benefits of food stamps and need for food stamps in their rural communities despite the stigma associated with accepting assistance from safety net programs (Andress and Fitch 2016; Mulik and Haynes‐Maslow 2017). Together, these findings on material hardship and safety net programs findings indicate a need for future research on the mechanisms of economic hardship in rural households, including material hardship, income, income transfers from safety net programs, debt, savings, and perceived financial stress.

Our findings on rural material hardship also demonstrate that other factors, yet to be identified, may protect rural households from material hardship. For example, informal and formal social supports may be more robust in rural areas, including borrowing money from friends or family and receiving food from a food bank or Supplemental Nutrition Assistance Program (Nolan et al. 2017).

The relationship between different forms of material hardship may also contribute to a household’s experience of hardship and should be studied in future research. To date, few researchers have examined the relationships between transportation hardship and food or healthcare access hardships, although lack of transportation is cited as a barrier to food and healthcare access (Syed et al. 2013; Walker et al. 2010). Further, access to healthcare coverage, for example, may reduce worry about paying for food, particularly in households with children (Kino et al. 2018). Likewise, households with food insecurity may be more likely to delay healthcare for children, including routine well‐childcare visits, medications, and sick care (Ma et al. 2008). This may be because parents experiencing material hardship need to prioritize the purchase of resources like food and medical care based on the most immediate need.

### Limitations and Conclusions

Our findings on the prevalence of U.S. material hardship may differ from other studies due to the hardships we calculated and our rural sample. Whereas our sample is generalizable to the general population of adults in Iowa, most researchers examine material hardship using nationally representative samples concentrated in urban areas (Conrad‐Hiebner and Paschall 2017), households with children (Gershoff et al. 2007; Rodems and Shaefer 2020), or low‐income households (Heflin 2016). Furthermore, as mentioned earlier, most rural scholars use measures of community size and population density (Tickamyer 2020). Our estimate of Iowa’s rural population (20 percent) is considerably smaller than the US Census Bureau’s estimate (36 percent), which is based on the Bureau’s delineation of urbanized areas (population of 50,000 or more) and urban clusters (population between 2,500 and fewer than 50,000) (see Table 1). The Bureau defines rural areas as those not encompassed within an urbanized area or urban cluster, that is, any area with a population below 2,500. Instead, our measure of rurality includes respondents who indicated living on a farm, in a rural area, or a rural subdivision outside of city limits. It is possible that our subjective measure of rurality carries measurement bias, which could affect the demonstrated relationship between rurality and material hardship.

Another limitation relates to our cross‐sectional survey design: Our data offers a snapshot of economic hardship and cannot address issues of causality or temporal ordering. Despite these limitations, our study contributes to the literature by investigating the relationship between rurality, material hardship, and income. Two in five households in our study of Iowans had material hardship, suggesting that material hardship in the US is more common than previously reported. Though higher incomes and ages, rurality, and White racial status buffered households from material hardship, these factors did not prevent material hardship among these groups. These findings indicate the importance of helping all households, regardless of income, meet their basic needs for food, medical care, and transportation.

Our final contribution pertains to rurality; contrary to our expectations, rurality buffered households from having more material hardship. In the future, researchers should seek to explain the mechanisms by which rurality influences the experience of material hardship. Identifying the underlying factors of rurality could lead to new efforts to prevent material hardship among our most vulnerable citizens and enhance their financial wellbeing.

## Conflict of Interest

None.

## Appendix A. Variables for the Logistic Regression Analyses

**Table:**

Construct	Survey Item	Scaling
Food Hardship		*If any of 5 indicators ≥ 1, then hardship =1*.
	The food I/we bought just didn’t last and I/we didn’t have money to get more	0 = never true
		1 = sometimes true
		2 = often true
	I/we couldn’t afford to eat balanced meals	0 = never true
		1 = sometimes true
		2 = often true
	Adults in the household cut the size of meals or skipped meals because there wasn’t enough money for food	0 = never true
		1 = sometimes true
		2 = often true
	I/we ate less then I/we should have because there wasn’t enough money to buy food	0 = no
		1 = yes
	I/we were hungry but didn’t eat because there wasn’t enough money for food	0 = no
		1 = yes
Transportation Hardship		*If (Number of vehicles – Number of adults) < 0, then hardship=1*.
	How many licensed vehicles were owned or available for regular use by members of your household during the last 12 months?	–
	How many adults, that is people over the age of 18, live in your household?	–
Healthcare Access Hardship		*If any of 4 indicators ≥ 1, then hardship =1*.
	Do you have any kind of health care coverage, including health insurance, prepaid plans such as HMOs, or government plans such as Medicaid or Medicare?	0 = no
		1 = yes
	In the past 12 months, was there a time when you needed routine wellness or preventive care but could not get it for any reason?	0 = no
		1 = yes
	In the past 12 months, was there any time when you needed transportation to or from a health care visit but could not get it for any reason?	0 = no
		1 = yes
	In the past 12 months, how often have you worried about your ability to pay for the cost of transportation to or from a health care visit?	0 = never
		1 = sometimes
		2 = usually
		3 = always
Overall Hardship		*If any Food, Transportation, or Healthcare Hardship = 1, then hardship = 1*.
